# Is thalamic deep brain stimulation the right target to improve laryngeal dystonia symptoms?

**DOI:** 10.3389/dyst.2025.15271

**Published:** 2025-09-29

**Authors:** Lena C. O’Flynn, Michael T. Barbe, Shiro Horisawa, Julie M. Barkmeier-Kraemer, Andrew Blitzer, Jeremy D. W. Greenlee, Farid Hamzei-Sichani, Paolo Moretti, Srikantan S. Nagarajan, Nutan Sharma, Shervin Rahimpour, Jan Rusz, Elina Tripoliti, Giovanni Battistella, Kristina Simonyan

**Affiliations:** 1 Department of Otolaryngology-Head and Neck Surgery, Massachusetts Eye and Ear and Harvard Medical School, Boston, MA, United States,; 2 Program in Speech Hearing Bioscience and Technology, Harvard University, Boston, MA, United States,; 3 University Hospital Cologne, Department of Neurology, Cologne, Germany,; 4 Department of Neurosurgery, Tokyo Women’s Medical University, Tokyo, Japan,; 5 Department of Communication Sciences and Disorders, The University of Utah, Salt Lake City, UT, United States,; 6 Department of Otolaryngology–Head and Neck Surgery, The University of Utah, Salt Lake City, UT, United States,; 7 Department of Otolaryngology-Head and Neck Surgery, Columbia University College of Physicians and Surgeons, New York, NY, United States,; 8 Department of Neurosurgery, University of Iowa, Iowa City, IA, United States,; 9 Department of Neurological Surgery, University of Massachusetts Medical School, UMass Memorial Healthcare, Worcester, MA, United States,; 10 Department of Neurology, The University of Utah, Salt Lake City, UT, United States,; 11 Department of Radiology and Biomedical Imaging, University of California San Francisco, San Francisco, CA, United States,; 12 Department of Neurology, Massachusetts General Hospital, Boston, MA, United States,; 13 Department of Neurosurgery, The University of Utah, Salt Lake City, UT, United States,; 14 Department of Circuit Theory, Faculty of Electrical Engineering, Czech Technical University in Prague, Prague, Czechia,; 15 Department of Clinical and Movement Neurosciences, Institute of Neurology, University College London, National Hospital for Neurology and Neurosurgery, UCLH NHS Trust, London, United Kingdom,; 16 Departments of Neurology and Neurolaryngology, Medical School, Athens University, Athens, Greece

**Keywords:** laryngeal dystonia, voice tremor, deep brain stimulation, clinical trial, neuromodulation

## Introduction

Laryngeal dystonia (LD), formerly known as spasmodic dysphonia, is one of the common forms of focal dystonia characterized by involuntary spasms in the laryngeal muscles that selectively impair speech production. The current standard of care for patients with LD is symptom management with botulinum injections (BoNT) into the affected laryngeal muscles [1]. However, BoNT is ineffective in nearly 40% of LD patients [2], and responders have benefits for only about 30% of each injection cycle [3]. Thus, developing effective, long-lasting therapeutic interventions for these patients is critical, as highlighted during the latest NIH workshops on research priorities in dystonia [1, 4].

The published article by Honey and colleagues [5] presents the first clinical trial assessing deep brain stimulation (DBS) of the ventral intermediate nucleus of the thalamus (Vim-DBS) as a treatment option for LD. The authors present six patients with LD and voice tremor (VT), who were recruited for the phase I prospective randomized double-blind cross-over trial to investigate the safety and efficacy of unilateral (left) Vim-DBS. Patients were randomized into two groups: one group received 3 months of DBS stimulation (DBS-ON) followed by 3 months of no stimulation (DBS-OFF), while the other group underwent the reverse sequence. The DBS outcomes relevant to voice symptoms were assessed using Voice-Related Quality of Life (V-RQOL), Voice Handicap Index (VHI), and the Unified Spasmodic Dysphonia Rating Scale (USDRS) questionnaires.

Given the lack of effective and long-lasting treatment options for these patients, this is an important area of study that requires rigorous methodological and statistical approaches. Our multidisciplinary team has critically assessed the reported findings and identified that the conclusions of this study should be interpreted with greater caution, taking into account the likely weight of this study’s outcomes on shaping future research and setting standards for the clinical care of these patients.

## Major findings and their interpretation

In this section, we highlight major findings and limitations of this study. First, the study was designed as a double-blind clinical trial, which is a standard for robust evaluation of treatment effects. However, the authors report that all patients became aware of the used blinded sequence during the study, rendering the study’s blinding unsuccessful. Moreover, it appears that patients were retrospectively asked to find out which group they were randomized to, as they “correctly guessed which blinded group they were in.” As this procedure is not defined in the study’s clinical protocol, the motivation for the additional retrospective unblinding is unclear. Second, the cross-over design did not include a wash-out period between DBS-ON and OFF conditions, likely contaminating the effects of each condition. These design failures are critical because they effectively reclassified the study as an open-label trial. Therefore, the authors’ presentation of this trial, followed by the discussion of its outcomes as if patients were part of a double-blind cross-over study design, is misleading. As a recommendation, adherence to the clinical trial protocol is imperative, as invasive procedures, such as DBS, are associated with a stronger placebo effect (over 50%) compared to non-invasive treatment options [6, 7].

Relevant to the symptom assessment as a primary outcome of Vim-DBS surgery, the symptom quantification measurements used in this study were not specific to capturing LD symptoms. The V-RQOL and VHI are patient-reported measures that assess the impact of voice on an individual’s wellbeing and quality of life. It is a well-known clinical observation that the quality of life and LD symptom severity do not necessarily correlate, as patients with mild symptoms may experience greater challenges with their quality of life than those with more severe symptoms. Furthermore, these measurements do not differentiate between disorder-specific aspects of voice symptoms, such as LD-characteristic voice breaks or VT-characteristic rhythmic oscillations. Similarly, the overall severity component of the USDRS is a compound score of multiple LD-specific (e.g., roughness, breathiness, strain) and VT measurements, without offering differential diagnostics. Because of the lack of specificity, these tools may inaccurately assess LD and VT symptom severity, and, therefore, more specialized assessments of LD-characteristic voice breaks, harshness/strain, breathiness, and tremor have been recommended for the quantification of LD and VT symptoms [1, 8, 9].

In terms of study outcomes, the authors state that “*every* patient reported an improvement in quality of life (*p* = 0.07) and had an improvement in quality of their voice (*p* = 0.06).” This statement contradicts the actual outcome of this clinical trial, given that one out of six patients did not experience any symptom improvement, thus pointing to an over-generalization of findings. Moreover, all clinical trials ought to strictly adhere to their pre-defined study protocol, including criteria for patient inclusion, statistical methods, and a priori set thresholds for outcome reporting that are published in clinicaltrials.gov prior to study initiation. The reported findings include a patient outside the study’s eligible ages and differ in the planned statistics, which were initially set to perform an analysis of variance (ANOVA), followed by Bonferroni-corrected pairwise comparisons.

Importantly, the reported findings did not reach statistical significance, indicating that there was no statistically significant improvement of LD voice symptoms following Vim-DBS. These results are similar to previous reports of the therapeutic efficacy of Vim-DBS in patients with LD and VT, which have also failed to show any statistically significant effects [10]. Therefore, the authors’ emphasis that the patients had improvement of their symptoms is not substantiated and should be viewed with great caution. While it is possible that non-significant outcomes were due to the study being underpowered, a discrepancy in responsiveness between LD and VT symptoms may have also contributed to the observed effect. To that end, although all patients were reported to have VT, and some had essential tremor (ET), this study did not address the effects of Vim-DBS on the patients’ VT symptoms. As such, it remains unclear to what degree the outcomes of this study, albeit statistically non-significant, were driven by the presence of VT symptoms and their response to DBS. Notably, the same group published the therapeutic effects of Vim-DBS in VT patients, showing that bilateral and unilateral Vim-DBS significantly reduces voice symptoms compared to baseline [11]. These results have since been replicated by other research groups [12–15]. Moreover, Vim-DBS has been shown to be highly effective in ameliorating other forms of tremor and has become the standard of care for drug-refractory ET since receiving FDA approval in 1997 [16, 17]. Thus, in light of the absence of classification of differences between LD and VT symptoms, the results of this study suggest that the reported changes in voice symptoms might have been more due to a reduction of VT than LD symptoms.

Unlike tremor, DBS of the globus pallidus pars interna (GPi-DBS) and the subthalamic nucleus (STN-DBS) are most effective in reducing dystonic symptoms, especially in patients with drug-refractory cervical, segmental, and generalized dystonias [18]. Clinical reports investigating the efficacy of GPi-DBS on LD symptoms without VT have shown some therapeutic efficacy with minimal adverse events [19, 20]. Specifically, Finger et al. performed a detailed auditory-perceptual evaluation of voice and speech in patients with dystonia following bilateral GPi-DBS, demonstrating significant improvements in voice quality parameters, including overall grade, roughness, and strain at 12 months post-surgery [21]. Their findings emphasize the delayed yet substantial therapeutic impact of GPi-DBS on voice symptoms, further reinforcing GPi as a suitable target for treating dystonic voice disorders. Similar long-term improvements in voice and speech functions following GPi-DBS were also observed in patients with segmental dystonia [22] and patients with Meige syndrome [23]. A recent comparative investigation between GPi- and Vim-DBS in patients with LD and co-occurring VT demonstrated that GPi-DBS was superior in reducing LD-characteristic voice breaks, continuous voicing, and overall speech intelligibility. In contrast, Vim-DBS was most effective in reducing VT intensity [24]. Collectively, these studies underscore that GPi-DBS is the scientifically justified neurosurgical target for dystonic voice disorders, compared to Vim.

Finally, interpreting the Vim-DBS outcome as a result of modulation of the cerebellar circuitry and relating the latter to the *primary* pathophysiology of LD and the neurophysiology of speech, in general, is not supported by either the findings of this clinical trial or the investigation of the cerebello-thalamic circuitry in these patients. It is notable that a subsequent study by the Honey group [25] used diffusion MRI tractography in the same six LD and VT patients to examine a stimulation “sweet spot” of benefit and define individual biomarkers of their Vim-DBS response. To do so, authors arbitrarily categorized individual DBS contacts as “effective” or “ineffective” in improving LD symptoms without reporting statistical or numerical thresholds as part of this classification. The results showed no statistically significant “sweet spot,” while the segmentation analysis found that “effective contacts” targeted thalamic areas linked to the sensorimotor region, and “ineffective contacts” targeted areas connected to the prefrontal region. These findings did not correlate with LD symptom improvement. Nevertheless, the study concluded that stimulation of thalamic sensorimotor areas is associated with improvement in LD symptoms and claimed to have identified a novel biomarker for DBS targeting. Although an important research question, these statements should be interpreted with caution as they neither identify the primary pathophysiological factors of LD nor define the neural correlates of the Vim-DBS response.

## Discussion and recommendations for future voice and speech DBS research

In this commentary, we argue that the negative results of this phase I Vim-DBS clinical trial in LD patients do not support the next phase II investigations of Vim-DBS in this disorder. Given the impact of brain surgery on one hand and the readiness of the vast majority of LD patients to enlist for new treatments on the other hand [26], the findings of this study should not be overinterpreted when designing new research studies or making clinical neurosurgical decisions for the treatment of LD. Future clinical trials in LD and other dystonias and movement disorders, in general, should adhere to the guidelines of Good Clinical Practice to ensure the proper design, conduct, analysis, reporting, and interpretation of the data in clinical trials. These are especially important when working with clinical populations that are desperate for treatment. Specifically, clinical trials must strictly adhere to the predetermined clinical trial design, including criteria for patient inclusion and exclusion, and the *a priori* set hypotheses, methodological analyses, and statistical thresholds. Furthermore, rigorous power analyses must be performed prior to study initiation to ensure an adequate sample size, thereby enhancing the study’s ability to detect a true effect and minimize statistical errors. This is imperative in voice and speech studies to control for symptom variability.

Voice and speech symptoms can be complex; therefore, clinicians and researchers should clearly define the type and form of speech impairment to be studied. In situations of co-occurring disorders, such as VT with LD, a clear distinction of symptoms and measurement tools must be identified. For a more robust understanding of the clinical symptoms, clinical trials should incorporate both clinician-objective (e.g., acoustic data) and patient-subjective (e.g., questionnaires) outcomes.

Lastly, in adherence to general policies for protected health information, open access to voice and speech material and the precise description of DBS electrode contact locations and stimulation parameters should be reported for all patients and trials for replicability purposes.
